# Supplementary material for: An apical junction protein antagonizes mechanosensitive calcium signaling to establish stochastic choices of olfactory neuron subtypes
Source: bioRxiv. 2026 Mar 16:2026.03.12.710951. Preprint. [Version 1] doi: 10.64898/2026.03.12.710951 (PMC13015320; doi:10.64898/2026.03.12.710951)
Supplement: Supplement 1 [file NIHPP2026.03.12.710951v1-supplement-1.pdf]

bioRxiv preprint doi: https://doi.org/10.64898/2026.03.12.710951; this version posted March 16, 2026. The copyright holder for this preprint (which was not certified by peer review) is the author/funder, who has granted bioRxiv a license to display the preprint in perpetuity. It is made available under aCC-BY 4.0 International license.

Supplemental Information

Supplemental Materials and Methods

Supplemental References

Supplemental Figure Legends

Supplemental Figures

Supplemental Materials and Methods

Strains and transgenes

Mutants

eri-1(mg366) IV; *lin-15b(n744)* X (Sieburth, Ch'ng et al. 2005)

slo-1(ky399gf) V (Troemel, Sagasti et al. 1999)

slo-1(ky389gf) V (Troemel, Sagasti et al. 1999)

slo-1(eg142lf) V (Kim, Pierce-Shimomura et al. 2009)

slo-2(ok2214lf) V (*C. elegans* knockout consortium)

ajm-1(vy11lf) X (this study)

ajm-1(ok160lf) X (Koppen, Simske et al. 2001)

unc-2(lj1lf) X (Tam, Mathews et al. 2000)

egl-19(n582rf) IV (Lee, Lobel et al. 1997)

egl-19(ad695gf) IV (Lee, Lobel et al. 1997)

del-1(ok150lf) X (*C. elegans* knockout consortium)

bioRxiv preprint doi: https://doi.org/10.64898/2026.03.12.710951; this version posted March 16, 2026. The copyright holder for this preprint (which was not certified by peer review) is the author/funder, who has granted bioRxiv a license to display the preprint in perpetuity. It is made available under aCC-BY 4.0 International license.

del-1(wy1014gf) X (Tao, Coakley et al. 2022)

Fluorescent protein knock-in strains

ajm-1a-m::ZF1::mNG knock-in (Figures 2, 6, S2, S3, S4)

ajm-1(vy359[ajm-1a-m::ZF1::mNG^3xFLAG]) X (this study)

Parent strain of *ajm-1(vy359)*: *ajm-1(vy356[ajm-1a-m::ZF1::mNG^SEC^3xFLAG])* X (this study)

ajm-1a,c-m::mNG knock-in (Figures 2, S2)

ajm-1(vy189[ajm-1a,c-m::mNG^3xFLAG]) X (this study)

Parent strain of *ajm-1(vy189)*: *ajm-1(vy175[ajm-1a,c-m::mNG^SEC^3xFLAG])* X (this study)

ajm-1a,d-m::ZF1::mNG knock-in (Figures 2, 6, S2, S4)

ajm-1(vy319[ajm-1a,d-m::ZF1::mNG^3xFLAG]) X (this study)

Parent strain of *ajm-1(vy319)*: *ajm-1(vy318[ajm-1a,d-m::ZF1::mNG^SEC^3xFLAG])* X (this study)

mNG::ajm-1b,c,d,f knock-in (Figures 2, S2)

ajm-1(vy314[mNG^3xFLAG::ajm-1b,c,d,f]) X (this study)

Parent strain of *ajm-1(vy314)*: *ajm-1(vy313[mNG^SEC^3xFLAG::ajm-1b,c,d,f])* X (this study)

bioRxiv preprint doi: https://doi.org/10.64898/2026.03.12.710951; this version posted March 16, 2026. The copyright holder for this preprint (which was not certified by peer review) is the author/funder, who has granted bioRxiv a license to display the preprint in perpetuity. It is made available under aCC-BY 4.0 International license.

ajm-1(vy11)a,c-m::mNG knock-in (Figure 2)

ajm-1(vy340[ajm-1(vy11)a,c-m::mNG^3xFLAG]) X (this study)

Parent strain of *ajm-1(vy340)*: *ajm-1(vy338[ajm-1(vy11)a,c-m::mNG^SEC^3xFLAG])* X (this study)

ajm-1a-m::GFP11 knock-in (Figures 2, S2)

ajm-1(vy434[ajm-1a-m::GFP11]) X (this study)

ajm-1a,c-m::TagRFP-T knock-in (Figures 3, S2)

ajm-1(vy438[ajm-1a,c-m::TagRFP-T^3xFLAG]) X (this study)

Parent strain of *ajm-1(vy438)*: *ajm-1(vy435[ajm-1a,c-m::TagRFP-T^SEC^3xFLAG])* X (this study)

ajm-1(vy11)a,c-m::TagRFP-T knock-in (Figure 3)

ajm-1(vy440[ajm-1(vy11)a,c-m::TagRFP-T^3xFLAG]) X (this study)

Parent strain of *ajm-1(vy440)*: *ajm-1(vy439[ajm-1(vy11)a,c-m::TagRFP^SEC^3xFLAG])* X (this study)

GFP::unc-70 knock-in (Figure 3)

bioRxiv preprint doi: https://doi.org/10.64898/2026.03.12.710951; this version posted March 16, 2026. The copyright holder for this preprint (which was not certified by peer review) is the author/funder, who has granted bioRxiv a license to display the preprint in perpetuity. It is made available under aCC-BY 4.0 International license.

unc-70(cas962[GFP::unc-70]) V (Jia, Li et al. 2019)

dlg-1::GFP knock-in (Figures 6 and S4)

ML2615 *dlg-1(mc103[dlg-1::GFP])* X (Cohen, Sparacio et al. 2020)

GFP::loxP::AID::let-413 knock-in (Figures 6 and S4)

BOX466 *let-413(mib81[GFP::loxP::AID::let-413])* X (Riga, Cravo et al. 2021)

slo-1::GFP knock-in (Figures S5 and S6)

slo-1(cim105[slo-1::GFP]) V (Oh, Haney et al. 2017)

del-1::GFP knock-in (Figure S7)

del-1(wy1149[del-1::GFP] X (Tao, Coakley et al. 2022)

<u>*Integrated transgenes*</u>

Integrated transgenes	Figures
vyIs68 [*str-2p::TagRFP; srsx-3p::GFP*] III (Cochella, Tursun et al. 2014)	1
kyIs140 [*str-2p::GFP; lin-15(+)*] I (Troemel, Sagasti et al. 1999)	1, 4, 5, 6, 7, S1, S2, S4, S6, S7
kyIs323 [*str-2::GFP; ofm-1::GFP*] II	1

bioRxiv preprint doi: https://doi.org/10.64898/2026.03.12.710951; this version posted March 16, 2026. The copyright holder for this preprint (which was not certified by peer review) is the author/funder, who has granted bioRxiv a license to display the preprint in perpetuity. It is made available under aCC-BY 4.0 International license.

jcIs1 [*ajm-1::GFP + unc-29(+) + rol-6(su1006)]* IV (Koppen, Simske et al.	2
vyIs56 [*odr-1p::TagRFP*] III (Cochella, Tursun et al. 2014)	2, S1
oxTi973 [*eft-3p::GFP::2xNLS::tbb-2 3' UTR + HygroR*] V (Frokjaer-Jensen, Davis et al. 2014)	3
vyIs96 [*str-2p::TagRFP*] V (this study)	6, S4, S7
heSi157 [*hsp-16.2p::Cre*] X (Gift from Sander van den Heuvel, Utrecht University)	6, S4, S6
otIs264 [*ceh-36p::tagRFP*] (Patel, Tursun et al. 2012)	S1
stIs10544 [*hlh-16::H1-wCherry::let-858 3' UTR*] (Murray, Boyle et al. 2012)	S5
vyIs97 [*str-2p::TagRFP*] IV (this study)	S6

<u>*Extrachromosomal arrays*</u>

Extrachromosomal arrays	Figures
vyEx1747, *1752* [*ajm-1 fosmid::GFP* (3 ng/µl); *ofm-1p::DsRed* (30 ng/µl)]	1, 2
vyEx2398, 2399 [*odr-3p::GFP1-10* (3 ng/µl); *ofm-1p::DsRed* (30 ng/µl)]	2
vyEx2423 [*ptr-10p::GFP1-10* (3 ng/µl); *ofm-1p::DsRed* (30 ng/µl)]	2, S2
vyEx2419 [*dpy-7p::GFP1-10* (3 ng/µl); *ofm-1p::DsRed* (30 ng/µl)]	2, S2
vyEx2203, 2204, 2206 [*odr-3p::ajm-1a e1-13::SL2::myrGFP* (7.5 ng/µl); *odr-1p::DsRed* (15 ng/µl); *ofm-1p::DsRed* (30 ng/µl)]	4
vyEx2207, 2208, 2209 [*rab-3p::ajm-1a e1-13::SL2::myrGFP* (7.5 ng/µl); *odr-1p::DsRed* (15 ng/µl); *ofm-1::DsRed* (30 ng/µl)]	4
vyEx2174, 2175, 2176 [*lin-26p::ajm-1a e1-13::SL2::myrGFP* (7.5 ng/µl); *odr-1p::DsRed* (15 ng/µl); *ofm-1::DsRed* (30 ng/µl)]	4
vyEx2251, 2252, 2254 [*ptr-10p:: ajm-1a e1-13::SL2::myrGFP* (7.5 ng/µl); *odr-1p::DsRed* (15 ng/µl); *ofm-1::DsRed* (30 ng/µl)]	4, 5
vyEx2200, 2201, 2202 [*dpy-7p::ajm-1a e1-13::SL2::myrGFP* (7.5 ng/µl); *odr-1p::DsRed* (15 ng/µl); *ofm-1::DsRed* (30 ng/µl)]	4
vyEx1958, 1959, 1961 [*ajm-1 fosmid::GFP* (3 ng/µl); *odr-1p::DsRed* (15 ng/µl); *ofm-1p::DsRed* (30 ng/µl)]	5

bioRxiv preprint doi: https://doi.org/10.64898/2026.03.12.710951; this version posted March 16, 2026. The copyright holder for this preprint (which was not certified by peer review) is the author/funder, who has granted bioRxiv a license to display the preprint in perpetuity. It is made available under aCC-BY 4.0 International license.

vyEx1954, 1955 [*lin-26p::ajm-1f* (7.5 ng/µl); *odr-1p::DsRed* (15 ng/µl); *ofm-1::DsRed* (30 ng/µl)]	5
vyEx2014-2016, 2217-2219 [*hsp-16.2p::zif-1* (50 ng/µl); *ofm-1p::DsRed* (30 ng/µl)]	6, S3, S4
vyEx2390, 2391 [*odr-3p::loxP::let-858 Term::sqt-1(d)::loxP::zif-1* (1 ng/µl); *egl-20p::GFP* (3 ng/µl)]	6, S4
vyEx2411, 2412, 2426 [*rab-3p::loxP::let-858 Term::sqt-1(d)::loxP::zif-1* (1 ng/µl); *egl-20p::2xnlsTagRFP* (12 ng/µl)]	6, S4
vyEx2416, 2499 [*ptr-10p::loxP::let-858 Term::sqt-1(d)::loxP::zif-1* (1 ng/µl); *egl-20p::GFP* (3 ng/µl)]	6, S4
vyEx2413-2415 [*dpy-7p::loxP::let-858 Term::sqt-1(d)::loxP::zif-1* (1 ng/µl); *egl-20p::GFP* (3 ng/µl)]	6, S4
vyEx2403-2407, 2409 [*dpy-7p::loxP::let-858 Term::sqt-1(d)::loxP:: GFP nanobody::zif-1* (1 ng/µl); *egl-20p::2xnlsTagRFP* (12 ng/µl)]	6, S4
vyEx2435-2440 [*ptr-10p::loxP::let-858 Term::sqt-1(d)::loxP:: GFP nanobody::zif-1* (1 ng/µl); *egl-20p::2xnlsTagRFP* (12 ng/µl)]	6
vyEx1684 [*slo-1p::2xnlsGFP* (5 ng/µl)] *(Alqadah, Hsieh et al. 2016)*	S5
vyEx2454, 2455, 2460 [*hsp-16.2p::GFP nanobody::zif-1* (20 ng/µl); *ofm-1p::DsRed* (30 ng/µl)]	S6
vyEx2457, 2458, 2464 [*odr-3p::GFP nanobody::zif-1* (7.5 ng/µl); *ofm-1p::DsRed* (30 ng/µl)]	S6
vyEx2461, 2462 [*dpy-7p::GFP nanobody::zif-1* (7.5 ng/µl); *ofm-1p::DsRed* (30 ng/µl)]	S6
vyEx2467, 2468, 2469 [*dpy-7p::loxP::let-858 Term::sqt-1(d)::loxP::GFPnanobody::zif-1* (1 ng/µl); *ofm-1p::DsRed* (30 ng/µl)]	S6
vyEx2470, 2471, 2472 [*ptr-10p::loxP::let-858 Term::sqt-1(d)::loxP::GFPnanobody::zif-1* (1 ng/µl); *ofm-1p::DsRed* (30 ng/µl)]	S6
vyEx2633, 2634, 2635 [*hsp-16.2p::GFP nanobody::zif-1* (20 ng/µl); *ofm-1p::DsRed* (30 ng/µl)]	S7
vyEx2641, 2642, 2643, 2644 [*odr-3p::GFP nanobody::zif-1* (7.5 ng/µl); *ofm-1p::DsRed* (30 ng/µl)]	S7
vyEx2645, 2646, 2647, 2648 [*rab-3p::GFP nanobody::zif-1* (7.5 ng/µl); *ofm-1p::DsRed* (30 ng/µl)]	S7

bioRxiv preprint doi: https://doi.org/10.64898/2026.03.12.710951; this version posted March 16, 2026. The copyright holder for this preprint (which was not certified by peer review) is the author/funder, who has granted bioRxiv a license to display the preprint in perpetuity. It is made available under aCC-BY 4.0 International license.

vyEx2792, 2793, 2794, 2795 [*ptr-10p::GFP nanobody::zif-1* (7.5 ng/µl); *ofm-1p::DsRed* (30 ng/µl)]	S7
vyEx2649, 2650, 2651, 2652 [*dpy-7p::GFP nanobody::zif-1* (7.5 ng/µl); *ofm-1p::DsRed* (30 ng/µl)]	S7

Plasmid construction

ajm-1b 662bp RNAi was generated by subcloning a 662 bp fragment (F: GTCTGGCATGAAAACAGAAATAATTTCTG; B: TGAAAATGTGTACGTGTAGGTTGTTC) containing the last 11 bp *ajm-1b* exon 9 and 651 bp of *ajm-1b 3' UTR* into the pAD12 vector (Dillin, Crawford et al. 2002).

ajm-1c RNAi was generated by subcloning a 397 bp fragment (F: GTTGTTGCATTATAAGCCACTTTTCAATC; B: GAATTTGATAGTTTTTATTTATTCAAAATTTGT TCATACATAAAC) containing the last 15 bp of the *ajm-1c* exon 13 and 382 bp of the *ajm-1c 3' UTR* into the pAD12 vector (Dillin, Crawford et al. 2002).

dlg-1 RNAi was generated by subcloning a 3462 bp fragment (F: AAGCGCATAAAGCGATAGAAAATGTG; B: CTAATGACGTGGCACCCAAATTG) spanning *dlg-1* exon 2 to 12 into the pAD12 vector (Dillin, Crawford et al. 2002).

let-413 RNAi was generated by subcloning a 4204 bp fragment (F: CGATCAACTTCATATATCGCCACAATG; B: TTAGGAAGCATACACGGGAGTAC) covering all *let-413* exons into the pAD12 vector (Dillin, Crawford et al. 2002).

bioRxiv preprint doi: https://doi.org/10.64898/2026.03.12.710951; this version posted March 16, 2026. The copyright holder for this preprint (which was not certified by peer review) is the author/funder, who has granted bioRxiv a license to display the preprint in perpetuity. It is made available under aCC-BY 4.0 International license.

eft-3p::Cas9::U6p::ajm-1a-m sgRNA was generated by inserting *ajm-1a-m sgRNA* (CATCCTCAACCCATACGTTA) into the *pDD162 eft-3p::Cas9::empty sgRNA* vector (Dickinson, Ward et al. 2013, Dickinson, Pani et al. 2015, Dickinson and Goldstein 2016) (Addgene #47549 from Bob Goldstein's lab) using the Q5 site-directed mutagenesis kit (New England Biolabs).

eft-3p::Cas9::U6p::ajm-1a,c-m sgRNA was generated by inserting *ajm-1a,c-m sgRNA* (ACAGGAAACGTCGAACCGAC) into the *pDD162 eft-3p::Cas9::empty sgRNA* vector (Dickinson, Ward et al. 2013, Dickinson, Pani et al. 2015, Dickinson and Goldstein 2016) (Addgene #47549 from Bob Goldstein's lab) using the Q5 site-directed mutagenesis kit (New England Biolabs).

eft-3p::Cas9::U6p::ajm-1a,d-m sgRNA was generated by inserting *ajm-1a,d-m sgRNA* (GATTCTGAATGGCTTCGTAG) into the *pDD162 eft-3p::Cas9::empty sgRNA* vector (Dickinson, Ward et al. 2013, Dickinson, Pani et al. 2015, Dickinson and Goldstein 2016) (Addgene #47549 from Bob Goldstein's lab) using the Q5 site-directed mutagenesis kit (New England Biolabs).

eft-3p::Cas9::U6p::ajm-1b,c,d,f sgRNA was generated by inserting *ajm-1b,c,d,f sgRNA* (GTCACAGAAAAGTCGTCCTC) into the *pDD162 eft-3p::Cas9::empty sgRNA* vector (Dickinson, Ward et al. 2013, Dickinson, Pani et al. 2015, Dickinson and Goldstein 2016) (Addgene #47549 from Bob Goldstein's lab) using the Q5 site-directed mutagenesis kit (New England Biolabs).

bioRxiv preprint doi: https://doi.org/10.64898/2026.03.12.710951; this version posted March 16, 2026. The copyright holder for this preprint (which was not certified by peer review) is the author/funder, who has granted bioRxiv a license to display the preprint in perpetuity. It is made available under aCC-BY 4.0 International license.

ajm-1a-m::ZF1^mNG ^SEC^3xFLAG was constructed by PCR amplifying a 1500 bp *ajm-1* 5' homology region (F: AGAAGAGAGAAAAACAATGGAACGTCTTGAAAG; B: GGATCCGTTTCTATTCAAATCATTCGGGTTG) and a 1531 bp *ajm-1* 3' homology region (F: AGCAGGTGAGACAACTTTCAGCATG; B: CCATACACTAAACATTTCAGCGTGTCTTACTG), and subcloning into the pDS676 ZF1^mNG^SEC^3xFLAG vector (a gift from Daniel Shaye) containing a 111 bp of ZF1 sequence encoding a PIE-1 C-C-C-H type zinc-finger domain tag (Armenti, Lohmer et al. 2014). The nucleotide of the PAM site of the construct was mutagenized to generate a silent mutation from CATCCTCAACCCATACGTTA <u>CGG</u> to CATCCTCAACCCATACGTTt <u>CtG</u> using the Q5 site-directed mutagenesis kit (New England Biolabs).

ajm-1a,c-m::mNG ^SEC^3xFLAG was constructed by PCR amplifying a 572 bp *ajm-1* 5' homology region (F: TTTCTATTTCCGGTCCTCGCAC; B: TAATGCAACAACCTGTCGGTTC) and a 545 bp *ajm-1* 3' homology region (F: TAAGCCACTTTTCAATCCCAATTACC; B: GAGGACTTCTAATGCTTTAGGGAGC), and subcloning into the pDD240 mNG^SEC^3xFLAG vector (Dickinson, Ward et al. 2013, Dickinson, Pani et al. 2015, Dickinson and Goldstein 2016). The nucleotide of the PAM site of the construct was mutagenized to generate a silent mutation from ACAGGAAACGTCGAACCGAC <u>AGG</u> to ACAGGAAACGTCGAACCGAC <u>AaG</u> using the Q5 site-directed mutagenesis kit (New England Biolabs).

bioRxiv preprint doi: https://doi.org/10.64898/2026.03.12.710951; this version posted March 16, 2026. The copyright holder for this preprint (which was not certified by peer review) is the author/funder, who has granted bioRxiv a license to display the preprint in perpetuity. It is made available under aCC-BY 4.0 International license.

ajm-1a,d-m::ZF1^mNG ^SEC^3xFLAG was constructed by PCR amplifying a 1500 bp *ajm-1* 5' homology region (F: TGCATACTAGCCGCACTTC; B: GACATTGTGTGCAAATTCTGAGAAG) and a 1518 bp *ajm-1* 3' homology region (F: TAGATATAAATTCATCTTCATTTTTATTATGAGCC; B: GAGTCAATGACCACCTCCG), and subcloning into the pDS676 mNG^SEC^3xFLAG vector (a gift from Daniel Shaye) containing ZF1 zinc-finger domain tag (Armenti, Lohmer et al. 2014). The nucleotide of the PAM site of the construct was mutagenized to generate a silent mutation from GATTCTGAATGGCTTCGTAG CGG to GATTCTGAATGGCTTCGTAG CGt using the Q5 site-directed mutagenesis kit (New England Biolabs).

ajm-1b,c,d,f::mNG ^SEC^3xFLAG was constructed by PCR amplifying a 1469 bp *ajm-1* 5' homology region (F: TTCTCCATCATCATTTTGCC; B: ACAATTCTGAAAAAAAAACCTTTAAAATTTTC) and a 1428 bp *ajm-1* 3' homology region (F: ATGGACATAGAAAATCTACAATCG; B: TCCTGTCAGTTTTACACATTAAG), and subcloning into the pDD268 mNG^SEC^3xFLAG vector (Dickinson, Ward et al. 2013, Dickinson, Pani et al. 2015, Dickinson and Goldstein 2016). The nucleotide of the PAM site of the construct was mutagenized to generate a silent mutation from GTCACAGAAAAGTCGTCCTC CGG to GTCACAGAAAAGTCGTCCTC CaG using the Q5 site-directed mutagenesis kit (New England Biolabs).

ajm-1a-m::GFP11 ssODN was a 194-nucleotide single-stranded oligodeoxyribonucleotide ordered from IDT; the complete sequences are as follows (*ajm-1* homology region in uppercase letters):

bioRxiv preprint doi: https://doi.org/10.64898/2026.03.12.710951; this version posted March 16, 2026. The copyright holder for this preprint (which was not certified by peer review) is the author/funder, who has granted bioRxiv a license to display the preprint in perpetuity. It is made available under aCC-BY 4.0 International license.

AATGACGAGAACAACCGTGATCGTCTTTACAACCCGAATGATTTGAATAGAAACGG ATCCGgaggaggatcccgtgaccacatggtccttcatgagtatgtaaatgctgctgggattacaggcggaggttctAGCAG GTGAGACAACTTTCAGCATGGTCTTCAATGCCCAACATAATACAAAAGTTGTTGCAG

ajm-1a,c-m::TagRFP-T ^SEC^3xFLAG was constructed by PCR amplifying a 1514 bp *ajm-1* 5' homology region (F: ACTCTGAATTCCGAACCAC; B: TAATGCAACAACCTGTCGGTTCG) and a 1500 bp *ajm-1* 3' homology region (F: TAAGCCACTTTTCAATCCCAATTACC; B: ACACTTGAACATTGGAATATAAGTTGTTTACC), and subcloning into the pDD284 TagRFP-T^SEC^3xFLAG vector (Dickinson, Ward et al. 2013, Dickinson, Pani et al. 2015, Dickinson and Goldstein 2016). The nucleotide of the PAM site of the construct was mutagenized to generate a silent mutation from ACAGGAAACGTCGAACCGAC AGG to ACAGGAAACGTCGAACCGAC AaG using the Q5 site-directed mutagenesis kit (New England Biolabs).

odr-3p::GFP1-10 was generated by subcloning a 648 bp GFP region containing the first 10 β-strands into a vector containing 2677 bp of the *odr-3* promoter (Roayaie, Crump et al. 1998).

ptr-10p::GFP1-10 was generated by subcloning a 648 bp GFP region containing the first 10 β-strands into a vector containing 300 bp of the *ptr-10* promoter (Yoshimura, Murray et al. 2008).

dpy-7p::GFP1-10 was generated by subcloning a 648 bp GFP region containing the first 10 β-strands into a vector containing 217 bp of the *dpy-7* promoter (Gilleard, Barry et al. 1997).

bioRxiv preprint doi: https://doi.org/10.64898/2026.03.12.710951; this version posted March 16, 2026. The copyright holder for this preprint (which was not certified by peer review) is the author/funder, who has granted bioRxiv a license to display the preprint in perpetuity. It is made available under aCC-BY 4.0 International license.

odr-3p::ajm-1a e1-13::SL2::myrGFP was generated by subcloning a 3155 bp of *ajm-1a* cDNA region spanning from the first ATG of *ajm-1a* exon 1 to the first 144 bp of *ajm-1a* exon 13 into a vector containing a 2677 bp of *odr-3* promoter (Roayaie, Crump et al. 1998) and 238 bp of SL2 trans-splicing sequence followed by 891 bp of myrGFP sequence.

rab-3p::ajm-1a e1-13::SL2::myrGFP was generated by subcloning a 3155 bp of *ajm-1a* cDNA region spanning from the first ATG of *ajm-1a* exon 1 to the first 144 bp of *ajm-1a* exon 13 into a vector containing a 4383 bp of *rab-3* promoter (Stefanakis, Carrera et al. 2015) and 238 bp of SL2 trans-splicing sequence followed by 891 bp of myrGFP sequence.

lin-26p::ajm-1a e1-13::SL2::myrGFP was generated by subcloning a 3155 bp of *ajm-1a* cDNA region spanning from the first ATG of *ajm-1a* exon 1 to the first 144 bp of *ajm-1a* exon 13 into a vector containing a 5000 bp of *lin-26* promoter (Labouesse, Sookhareea et al. 1994, Labouesse, Hartwieg et al. 1996, Landmann, Quintin et al. 2004) and 238 bp of SL2 trans-splicing sequence followed by 891 bp of myrGFP sequence.

lin-26p::ajm-1f was generated by subcloning 5715 bp of *ajm-1f* cDNA into a vector containing 5000 bp of the *lin-26* promoter (Labouesse, Sookhareea et al. 1994, Labouesse, Hartwieg et al. 1996, Landmann, Quintin et al. 2004).

ptr-10p::ajm-1a e1-13::SL2::myrGFP was generated by subcloning a 3155 bp of *ajm-1a* cDNA region spanning from the first ATG of *ajm-1a* exon 1 to the first 144 bp of *ajm-1a* exon 13 into a

bioRxiv preprint doi: https://doi.org/10.64898/2026.03.12.710951; this version posted March 16, 2026. The copyright holder for this preprint (which was not certified by peer review) is the author/funder, who has granted bioRxiv a license to display the preprint in perpetuity. It is made available under aCC-BY 4.0 International license.

vector containing a 300 bp of *ptr-10* promoter (Yoshimura, Murray et al. 2008) and 238 bp of SL2 trans-splicing sequence followed by 891 bp of myrGFP sequence.

dpy-7p::ajm-1a e1-13::SL2::myrGFP was generated by subcloning a 3155 bp of *ajm-1a* cDNA region spanning from the first ATG of *ajm-1a* exon 1 to the first 144 bp of *ajm-1a* exon 13 into a vector containing a 217 bp of *dpy-7* promoter (Gilleard, Barry et al. 1997) and 238 bp of SL2 trans-splicing sequence followed by 891 bp of myrGFP sequence.

hsp-16.2p::zif-1 was generated by cloning 1744 bp of *zif-1* genomic DNA (Armenti, Lohmer et al. 2014) into a vector containing 394 bp of the *hsp-16.2* promoter (Bacaj and Shaham 2007).

odr-3p::loxP::let-858 Term::sqt-1(d)::loxP::zif-1 was generated by cloning 1744 bp of *zif-1* genomic DNA (Armenti, Lohmer et al. 2014) after a *loxP*-flanking region that contains 381 bp of the let-858 terminator region and a *sqt-1* dominant marker (Dickinson, Pani et al. 2015) into a vector containing 2677 bp of the *odr-3* promoter (Roayaie, Crump et al. 1998).

rab-3p::loxP::let-858 Term::sqt-1(d)::loxP::zif-1 was generated by cloning 1744 bp of *zif-1* genomic DNA (Armenti, Lohmer et al. 2014) after a *loxP*-flanking region that contains a 381 bp let-858 terminator region and a *sqt-1* dominant marker (Dickinson, Pani et al. 2015) into a vector containing 4383 bp of the *rab-3* promoter (Stefanakis, Carrera et al. 2015).

ptr-10p::loxP::let-858 Term::sqt-1(d)::loxP::zif-1 was generated by cloning 1744 bp of *zif-1* genomic DNA (Armenti, Lohmer et al. 2014) after a *loxP*-flanking region that contains a 381 bp

bioRxiv preprint doi: https://doi.org/10.64898/2026.03.12.710951; this version posted March 16, 2026. The copyright holder for this preprint (which was not certified by peer review) is the author/funder, who has granted bioRxiv a license to display the preprint in perpetuity. It is made available under aCC-BY 4.0 International license.

let-858 terminator region and a *sqt-1* dominant marker (Dickinson, Pani et al. 2015) into a vector containing 300 bp of the *ptr-10* promoter (Yoshimura, Murray et al. 2008).

dpy-7p::loxP::let-858 Term::sqt-1(d)::loxP::zif-1 was generated by cloning 1744 bp of *zif-1* genomic DNA (Armenti, Lohmer et al. 2014) after a *loxP*-flanking region that contains 381 bp of the let-858 terminator region and a *sqt-1* dominant marker (Dickinson, Pani et al. 2015) into a vector containing 217 bp of the *dpy-7* promoter (Gilleard, Barry et al. 1997).

hsp-16.2p::GFP nanobody::zif-1 was generated by cloning a 351 bp GFP nanobody fused with 1744 bp of *zif-1* genomic DNA (Wang, Tang et al. 2017) into a vector containing 394 bp of the *hsp-16.2* promoter (Bacaj and Shaham 2007).

dpy-7p::loxP::let-858 Term::sqt-1(d)::loxP::GFP nanobody::zif-1 was generated by cloning 351 bp GFP nanobody fused with 1744 bp of *zif-1* genomic DNA (Wang, Tang et al. 2017) after a *loxP*-flanking region that contains a 381 bp let-858 terminator region and a *sqt-1* dominant marker (Dickinson, Pani et al. 2015) into a vector containing 217 bp of the *dpy-7* promoter (Gilleard, Barry et al. 1997).

ptr-10p::loxP::let-858 Term::sqt-1(d)::loxP::GFP nanobody::zif-1 was generated by cloning 351 bp GFP nanobody fused with 1744 bp of *zif-1* genomic DNA (Wang, Tang et al. 2017) after a *loxP*-flanking region that containing a 381 bp let-858 terminator region and a *sqt-1* dominant markers (Dickinson, Pani et al. 2015) into a vector containing 300 bp of the *ptr-10* promoter (Yoshimura, Murray et al. 2008).

bioRxiv preprint doi: https://doi.org/10.64898/2026.03.12.710951; this version posted March 16, 2026. The copyright holder for this preprint (which was not certified by peer review) is the author/funder, who has granted bioRxiv a license to display the preprint in perpetuity. It is made available under aCC-BY 4.0 International license.

odr-3p::GFP nanobody::zif-1 was generated by cloning a 351 bp GFP nanobody fused with 1744 bp of *zif-1* genomic DNA (Wang, Tang et al. 2017) into a vector containing 2677 bp of the *odr-3* promoter (Roayaie, Crump et al. 1998).

rab-3p::GFP nanobody::zif-1 was generated by cloning a 351 bp GFP nanobody fused with 1744 bp of *zif-1* genomic DNA (Wang, Tang et al. 2017) into a vector containing 4383 bp of the *rab-3* promoter (Roayaie, Crump et al. 1998).

ptr-10p::GFP nanobody::zif-1 was generated by cloning a 351 bp GFP nanobody fused with 1744 bp of *zif-1* genomic DNA (Wang, Tang et al. 2017) into a vector containing 300 bp of the *ptr-10* promoter (Roayaie, Crump et al. 1998).

dpy-7::GFP nanobody::zif-1 was generated by cloning 351 bp GFP nanobody fused with 1744 bp of *zif-1* genomic DNA (Wang, Tang et al. 2017) into a vector containing 217 bp of the *dpy-7* promoter (Roayaie, Crump et al. 1998).

Supplemental References

Alqadah, A., Y. W. Hsieh, J. A. Schumacher, X. Wang, S. A. Merrill, G. Millington, B. Bayne, E. M. Jorgensen and C. F. Chuang (2016). "SLO BK Potassium Channels Couple Gap Junctions to Inhibition of Calcium Signaling in Olfactory Neuron Diversification." PLoS Genet **12**(1): e1005654.

bioRxiv preprint doi: https://doi.org/10.64898/2026.03.12.710951; this version posted March 16, 2026. The copyright holder for this preprint (which was not certified by peer review) is the author/funder, who has granted bioRxiv a license to display the preprint in perpetuity. It is made available under aCC-BY 4.0 International license.

Armenti, S. T., L. L. Lohmer, D. R. Sherwood and J. Nance (2014). "Repurposing an endogenous degradation system for rapid and targeted depletion of C. elegans proteins." Development **141**(23): 4640–4647.

Bacaj, T. and S. Shaham (2007). "Temporal control of cell-specific transgene expression in Caenorhabditis elegans." Genetics **176**(4): 2651–2655.

Cochella, L., B. Tursun, Y. W. Hsieh, S. Galindo, R. J. Johnston, C. F. Chuang and O. Hobert (2014). "Two distinct types of neuronal asymmetries are controlled by the Caenorhabditis elegans zinc finger transcription factor die-1." Genes Dev **28**(1): 34–43.

Cohen, J. D., A. P. Sparacio, A. C. Belfi, R. Forman-Rubinsky, D. H. Hall, H. Maul-Newby, A. R. Frand and M. V. Sundaram (2020). "A multi-layered and dynamic apical extracellular matrix shapes the vulva lumen in Caenorhabditis elegans." Elife **9**.

Dickinson, D. J. and B. Goldstein (2016). "CRISPR-Based Methods for Caenorhabditis elegans Genome Engineering." Genetics **202**(3): 885–901.

Dickinson, D. J., A. M. Pani, J. K. Heppert, C. D. Higgins and B. Goldstein (2015). "Streamlined Genome Engineering with a Self-Excising Drug Selection Cassette." Genetics **200**(4): 1035–1049.

Dickinson, D. J., J. D. Ward, D. J. Reiner and B. Goldstein (2013). "Engineering the Caenorhabditis elegans genome using Cas9-triggered homologous recombination." Nat Methods **10**(10): 1028–1034.

Dillin, A., D. K. Crawford and C. Kenyon (2002). "Timing requirements for insulin/IGF-1 signaling in C. elegans." Science **298**(5594): 830–834.

Frokjaer-Jensen, C., M. W. Davis, M. Sarov, J. Taylor, S. Flibotte, M. LaBella, A. Pozniakovsky, D. G. Moerman and E. M. Jorgensen (2014). "Random and targeted transgene

bioRxiv preprint doi: https://doi.org/10.64898/2026.03.12.710951; this version posted March 16, 2026. The copyright holder for this preprint (which was not certified by peer review) is the author/funder, who has granted bioRxiv a license to display the preprint in perpetuity. It is made available under aCC-BY 4.0 International license.

insertion in Caenorhabditis elegans using a modified Mos1 transposon." Nat Methods **11**(5): 529–534.

Gilleard, J. S., J. D. Barry and I. L. Johnstone (1997). "cis regulatory requirements for hypodermal cell-specific expression of the Caenorhabditis elegans cuticle collagen gene dpy-7." Mol Cell Biol **17**(4): 2301–2311.

Jia, R., D. Li, M. Li, Y. Chai, Y. Liu, Z. Xie, W. Shao, C. Xie, L. Li, X. Huang, L. Chen, W. Li and G. Ou (2019). "Spectrin-based membrane skeleton supports ciliogenesis." PLoS Biol **17**(7): e3000369.

Kim, H., J. T. Pierce-Shimomura, H. J. Oh, B. E. Johnson, M. B. Goodman and S. L. McIntire (2009). "The dystrophin complex controls bk channel localization and muscle activity in Caenorhabditis elegans." PLoS Genet **5**(12): e1000780.

Koppen, M., J. S. Simske, P. A. Sims, B. L. Firestein, D. H. Hall, A. D. Radice, C. Rongo and J. D. Hardin (2001). "Cooperative regulation of AJM-1 controls junctional integrity in Caenorhabditis elegans epithelia." Nat Cell Biol **3**(11): 983–991.

Labouesse, M., E. Hartwieg and H. R. Horvitz (1996). "The Caenorhabditis elegans LIN-26 protein is required to specify and/or maintain all non-neuronal ectodermal cell fates." Development **122**(9): 2579–2588.

Labouesse, M., S. Sookhareea and H. R. Horvitz (1994). "The Caenorhabditis elegans gene lin-26 is required to specify the fates of hypodermal cells and encodes a presumptive zinc-finger transcription factor." Development **120**(9): 2359–2368.

Landmann, F., S. Quintin and M. Labouesse (2004). "Multiple regulatory elements with spatially and temporally distinct activities control the expression of the epithelial differentiation gene lin-26 in C. elegans." Dev Biol **265**(2): 478–490.

bioRxiv preprint doi: https://doi.org/10.64898/2026.03.12.710951; this version posted March 16, 2026. The copyright holder for this preprint (which was not certified by peer review) is the author/funder, who has granted bioRxiv a license to display the preprint in perpetuity. It is made available under aCC-BY 4.0 International license.

Lee, R. Y., L. Lobel, M. Hengartner, H. R. Horvitz and L. Avery (1997). "Mutations in the alpha1 subunit of an L-type voltage-activated Ca2+ channel cause myotonia in Caenorhabditis elegans." EMBO J **16**(20): 6066–6076.

Murray, J. I., T. J. Boyle, E. Preston, D. Vafeados, B. Mericle, P. Weisdepp, Z. Zhao, Z. Bao, M. Boeck and R. H. Waterston (2012). "Multidimensional regulation of gene expression in the C. elegans embryo." Genome Research **22**(7): 1282–1294.

Oh, K. H., J. J. Haney, X. Wang, C. F. Chuang, J. E. Richmond and H. Kim (2017). "ERG-28 controls BK channel trafficking in the ER to regulate synaptic function and alcohol response in C. elegans." Elife **6**.

Patel, T., B. Tursun, Dylan P. Rahe and O. Hobert (2012). "Removal of Polycomb Repressive Complex 2 Makes C. elegans Germ Cells Susceptible to Direct Conversion into Specific Somatic Cell Types." Cell Reports **2**(5): 1178–1186.

Riga, A., J. Cravo, R. Schmidt, H. R. Pires, V. G. Castiglioni, S. van den Heuvel and M. Boxem (2021). "Caenorhabditis elegans LET-413 Scribble is essential in the epidermis for growth, viability, and directional outgrowth of epithelial seam cells." PLoS Genet **17**(10): e1009856.

Roayaie, K., J. G. Crump, A. Sagasti and C. I. Bargmann (1998). "The G alpha protein ODR-3 mediates olfactory and nociceptive function and controls cilium morphogenesis in C. elegans olfactory neurons." Neuron **20**(1): 55–67.

Sieburth, D., Q. Ch'ng, M. Dybbs, M. Tavazoie, S. Kennedy, D. Wang, D. Dupuy, J. F. Rual, D. E. Hill, M. Vidal, G. Ruvkun and J. M. Kaplan (2005). "Systematic analysis of genes required for synapse structure and function." Nature **436**(7050): 510–517.

Stefanakis, N., I. Carrera and O. Hobert (2015). "Regulatory Logic of Pan-Neuronal Gene Expression in C. elegans." Neuron **87**(4): 733–750.

bioRxiv preprint doi: https://doi.org/10.64898/2026.03.12.710951; this version posted March 16, 2026. The copyright holder for this preprint (which was not certified by peer review) is the author/funder, who has granted bioRxiv a license to display the preprint in perpetuity. It is made available under aCC-BY 4.0 International license.

Tam, T., E. Mathews, T. P. Snutch and W. R. Schafer (2000). "Voltage-gated calcium channels direct neuronal migration in Caenorhabditis elegans." Dev Biol **226**(1): 104–117.

Tao, L., S. Coakley, R. Shi and K. Shen (2022). "Dendrites use mechanosensitive channels to proofread ligand-mediated neurite extension during morphogenesis." Dev Cell **57**(13): 1615–1629 e1613.

Troemel, E. R., A. Sagasti and C. I. Bargmann (1999). "Lateral signaling mediated by axon contact and calcium entry regulates asymmetric odorant receptor expression in C. elegans." Cell **99**(4): 387–398.

Wang, S., N. H. Tang, P. Lara-Gonzalez, Z. Zhao, D. K. Cheerambathur, B. Prevo, A. D. Chisholm, A. Desai and K. Oegema (2017). "A toolkit for GFP-mediated tissue-specific protein degradation in C. elegans." Development **144**(14): 2694–2701.

Yoshimura, S., J. I. Murray, Y. Lu, R. H. Waterston and S. Shaham (2008). "mls-2 and vab-3 Control glia development, hlh-17/Olig expression and glia-dependent neurite extension in C. elegans." Development **135**(13): 2263–2275.

Supplemental Figure Legends

Figure S1. Genetic interactions of *ajm-1(vy11)* with *slo-1(gf)* in AWC asymmetry

(A) Double mutant analysis of *ajm-1(vy11)* with *slo-1(ky399gf)* mutants. Animals were scored at the adult stage. n, total number of animals scored.

(B) Quantification of general AWC identity phenotypes in wild type and *ajm-1(vy11)* mutants. Animals were scored at the adult stage. n, total number of animals scored.

Figure S2. Gene structure and analyses of *ajm-1* knock-in with various fluorescent tags

bioRxiv preprint doi: https://doi.org/10.64898/2026.03.12.710951; this version posted March 16, 2026. The copyright holder for this preprint (which was not certified by peer review) is the author/funder, who has granted bioRxiv a license to display the preprint in perpetuity. It is made available under aCC-BY 4.0 International license.

(**A**) Genomic structure of all *ajm-1* isoforms a-m with the location of CRISPR/Cas9 knock-in tags, mNG, TagRFP-T, or ZF1::mNG. RNAi target regions of different *ajm-1* isoforms are indicated in blue lines.

(B) Expression of the AWC^{ON} markers in adults. n, total number of animals scored. The AWC phenotype data of the wild type (row a) and *ajm-1(vy11)* (row b) are the same as those of Figure 1B (rows a and b, respectively).

(**C**, **D**) Tissue-specific visualization of endogenous AJM-1 protein expression in 3-fold stage embryos using split GFP in specific tissues. GFP11 is knocked in all AJM-1 isoforms a-m. GFP1-10 is under the control of a tissue-specific promoter expressed in pan-glial cells (C) or hypodermal cells (D). Scale bars, 5 µm

Figure S3. AJM-1a-m::ZF1::mNG is degraded upon heat-shock treatment

(**A**, **B**) Images of *ajm-1(vy359[ajm-1a-m::ZF1::mNG knock-in])* 1.5-fold embryo without **(**A**)** and with **(**B**)** *hsp-16.2p::zif-1* transgene after heat-shock treatment at 34 °C for 30 min. Images were taken 1.5 hrs after the heat-shock treatment finished. Scale bars, 5 µm.

Figure S4. *ajm-1* acts embryonically to promote AWC^{ON}

(A) Quantification of AWC asymmetry phenotypes in *ajm-1a-m::ZF1::mNG knock-in* (all AJM-1 isoforms tagged), *ajm-1a,d-m::ZF1::mNG knock-in* (AJM-1 isoform b and c not tagged), *dlg-1::GFP knock-in,* and *GFP::AID::let-413 knock-in* animals with heat-shock treatment in the absence of a tissue-specific knockdown transgenic array, or without heat shock in the presence of a transgenic array. +, heat-shock induction of endogenous protein knockdown at mixed embryonic stages. –, no heat-shock treatment. Adult animals from one or two independent lines

bioRxiv preprint doi: https://doi.org/10.64898/2026.03.12.710951; this version posted March 16, 2026. The copyright holder for this preprint (which was not certified by peer review) is the author/funder, who has granted bioRxiv a license to display the preprint in perpetuity. It is made available under aCC-BY 4.0 International license.

(combined) of each transgene were scored. n, total number of animals scored. The AWC phenotype data of the wild type (row a) and *ajm-1(vy11)* (row b) are the same as those of Figure 1B (rows a and b, respectively).

(B) Quantification of AWC asymmetry phenotypes in animals with knockdown of endogenous AJM-1 protein isoforms a-m containing *ZF1::mNG knock-in* at different developmental stages. Transgenic line expressing ZIF-1 (the substrate-binding subunit of the E3 ligase) from the heat-inducible promoter, *hsp-16.2p*, was scored. Embryos and larvae at different stages were collected and heat-shocked. HAE, hours after egg laying. Animals were scored at the adult stage. L1, first-stage larva. L2, second-stage larva. L3, third-stage larva. L4, fourth-stage larva. n, total number of animals scored.

(**C**) Timeline of *C. elegans* developmental events at 22°C. Eggs are laid about 150 minutes after fertilization. Gastrulation occurs 140-330 minutes after fertilization. AWC neurons are born at about 300 min, and their axons extend at about 450 min after fertilization. Embryos reach the 1.5-, 2-, and 3-fold stages at about 460 min, 490 min, and 550 min after fertilization, respectively. Animals hatch at about 840 minutes after fertilization. AWC asymmetry is established during embryogenesis and is maintained throughout the animal's life span.

Figure S5. *slo-1* expression is decreased in *ajm-1(vy11)* mutants

(A) Images of 1.5-fold stage embryos from wild type (non-transgenic), *slo-1(cim105[slo-1::GFP knock-in])*, and *slo-1(cim105[slo-1::GFP knock-in]); ajm-1(vy11).* Scale bar, 10 μm.

(B) Left panels: Images of *slo-1(cim105[slo-1::GFP knock-in])* 1.5-fold stage embryos immunostained with an anti-GFP antibody in wild type and *ajm-1(vy11)*. Right panel:

bioRxiv preprint doi: https://doi.org/10.64898/2026.03.12.710951; this version posted March 16, 2026. The copyright holder for this preprint (which was not certified by peer review) is the author/funder, who has granted bioRxiv a license to display the preprint in perpetuity. It is made available under aCC-BY 4.0 International license.

Quantification of anti-GFP immunostaining intensity in whole embryos and AWC. A stack of focal planes was projected with maximum intensity and compared for fluorescence intensity.

(C) Left panels: Images of wild-type and *ajm-1(vy11)* 1.5-fold stage embryos expressing *slo-1p::2xnlsGFP* in the nucleus. Right panel: Quantification of *slo-1p::2xnlsGFP* expression in whole embryos and AWC. The single focal plane with the brightest GFP fluorescence in the AWC at the top of the acquired image stacks was selected and compared for fluorescence intensity of the AWC nucleus.

(B, C) Insets, indicated by dashed boxes, are magnified by 2-fold. Arrows indicate the AWC nuclei. The AWC nucleus expressing *hlh-16p::H1-wCherry* is outlined with dashed lines. Scale bar, 10 µm. n, total number of animals quantified. Student's *t*-test was used for statistical analysis. Error bars, standard error of the mean. AU, arbitrary unit.

Figure S6. *slo-1* acts cell autonomously to promote the AWC^{ON} subtype

(A) Quantification of AWC asymmetry phenotypes in animals with knockdown of endogenous SLO-1 protein containing *GFP* knock-in, in specific tissues. Two or three independent lines expressing GFP nanobody::ZIF-1 from the heat-inducible promoter, *hsp-16.2p*, hypodermis-specific promoter, *dpy-7p*, or AWC-specific promoter, *odr-3p*, were scored. +, heat-shock induction of endogenous protein knockdown at mixed embryonic stages. –, no heat shock treatment. Animals were scored at the adult stage. n, total number of animals scored. Statistical comparisons between individual data groups e-i versus d were determined by a *Z*-test. Asterisks indicate comparisons that are different at $p < 0.05$. ns, not significant. The AWC phenotype data of the wild type (row a) are the same as those of Figure 1B (row a).

bioRxiv preprint doi: https://doi.org/10.64898/2026.03.12.710951; this version posted March 16, 2026. The copyright holder for this preprint (which was not certified by peer review) is the author/funder, who has granted bioRxiv a license to display the preprint in perpetuity. It is made available under aCC-BY 4.0 International license.

(B) Quantification of AWC asymmetry phenotypes in animals with knockdown of endogenous SLO-1 protein containing *GFP* knock-in at different developmental stages. Transgenic line expressing ZIF-1 from the heat-inducible promoter, *hsp-16.2p*, was scored. Mixed-stage embryos were heat-shocked at various stages. HAE, hours after egg laying. Animals were scored at the adult stage. n, total number of animals scored.

Figure S7. *del-1* acts both cell and non-cell autonomously in AWC asymmetry

Quantification of AWC asymmetry phenotypes in animals with knockdown of endogenous DEL-1 protein containing *GFP* knock-in in specific tissues. Four independent lines expressing GFP nanobody::ZIF-1 from the heat-inducible promoter, *hsp-16.2p*, AWC-specific promoter, *odr-3p*, pan-neuronal promoter, *rab-3p*, pan-glial cell-specific promoter, *ptr-10p*, or hypodermis-specific promoter, *dpy-7p*, were scored. +, heat-shock induction of endogenous protein knockdown at mixed embryonic stages. –, no heat shock treatment. Animals were scored at the adult stage. n, total number of animals scored. Statistical comparisons were made by a *Z*-test. Asterisks indicate comparisons that are different at $p < 0.05$. ns, not significant. The AWC phenotype data of the wild type (row a) and *ajm-1(vy11)* (row b) are the same as those of Figure 1B (rows a and b, respectively). The AWC phenotype data of the *del-1(ok150lf)* (row c) and *vy11 ok150* (row d) are the same as those of Figure 7 (rows b and u, respectively).

Figure S1

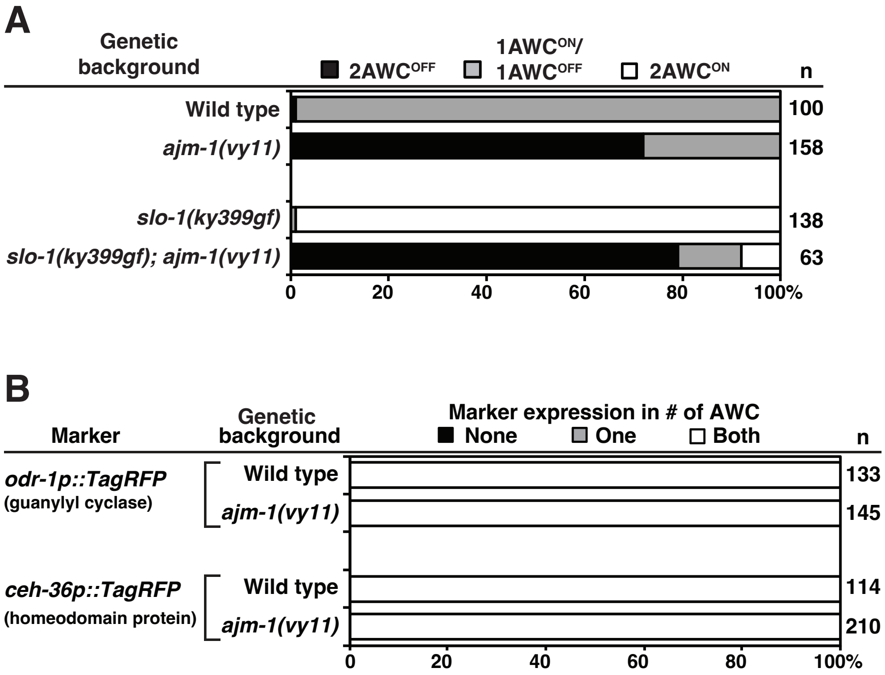
A
Genetic background
2AWC^OFF
1AWC^ON/ 1AWC^OFF
2AWC^ON
n
Wild type
ajm-1(vy11)
slo-1(ky399gf)
slo-1(ky399gf); ajm-1(vy11)
100
158
138
63
0
20
40
60
80
100%
B
Marker
Genetic background
Marker expression in # of AWC
None
One
Both
n
odr-1p::TagRFP
(guanylyl cyclase)
ceh-36p::TagRFP
(homeodomain protein)
Wild type
ajm-1(vy11)
Wild type
ajm-1(vy11)
133
145
114
210
0
20
40
60
80
100%

Figure S2

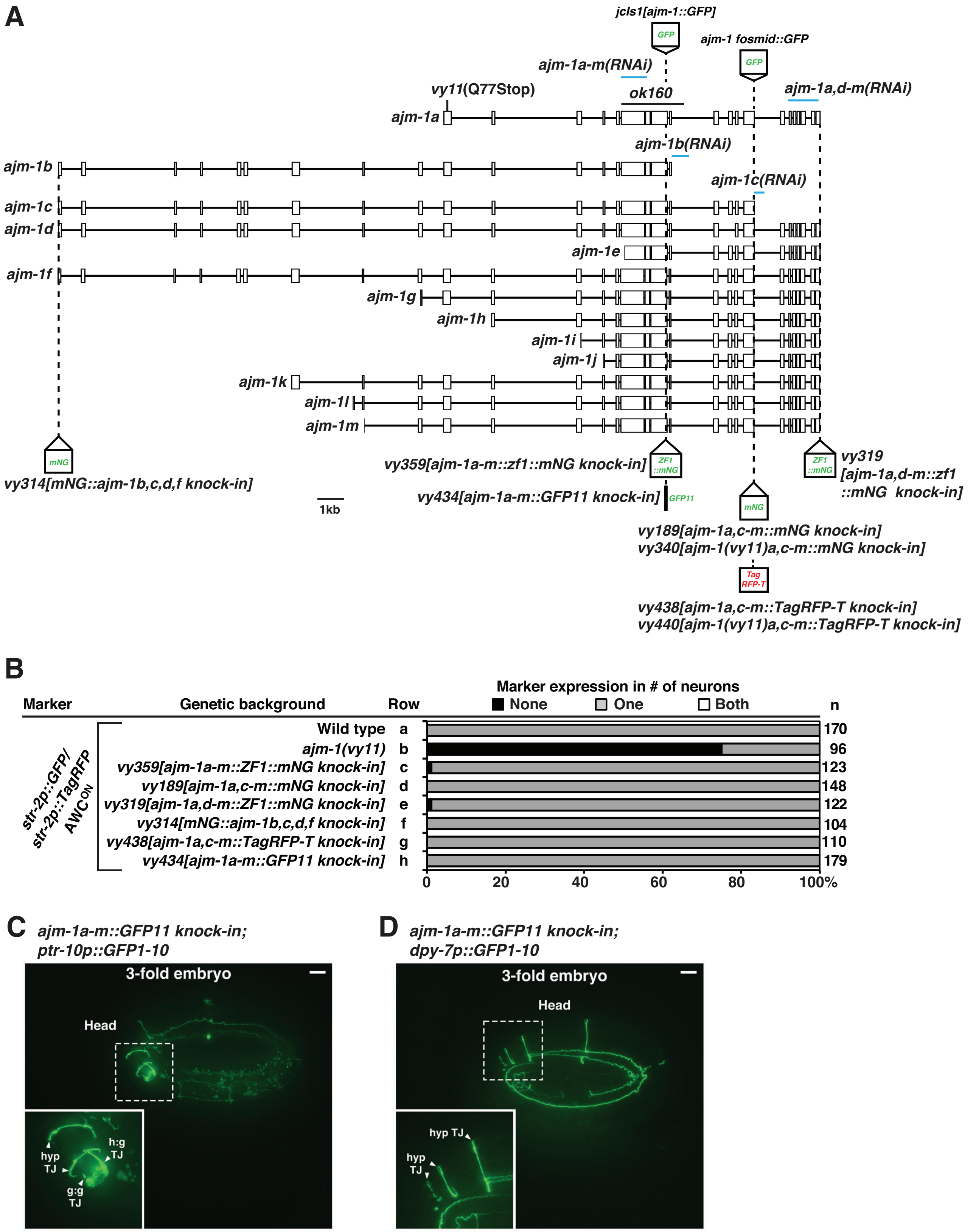
A
jcIs1[ajm-1::GFP]
GFP
ajm-1 fosmid::GFP
GFP
ajm-1a-m(RNAi)
vy11(Q77Stop)
ok160
ajm-1a,d-m(RNAi)
ajm-1a
ajm-1b(RNAi)
ajm-1b
ajm-1c(RNAi)
ajm-1c
ajm-1d
ajm-1e
ajm-1f
ajm-1g
ajm-1h
ajm-1i
ajm-1j
ajm-1k
ajm-1l
ajm-1m
mNG
vy314[mNG::ajm-1b,c,d,f knock-in]
ZF1 ::mNG
vy359[ajm-1a-m::zf1::mNG knock-in]
ZF1 ::mNG
vy319 [ajm-1a,d-m::zf1 ::mNG knock-in]
1kb
vy434[ajm-1a-m::GFP11 knock-in]
GFP11
mNG
vy189[ajm-1a,c-m::mNG knock-in]
vy340[ajm-1(vy11)a,c-m::mNG knock-in]
Tag RFP-T
vy438[ajm-1a,c-m::TagRFP-T knock-in]
vy440[ajm-1(vy11)a,c-m::TagRFP-T knock-in]
B
Marker
Genetic background
Row
Marker expression in # of neurons
None
One
Both
n
str-2p::GFP/ str-2p::TagRFP AWC^ON
Wild type a 170
ajm-1(vy11) b 96
vy359[ajm-1a-m::ZF1::mNG knock-in] c 123
vy189[ajm-1a,c-m::mNG knock-in] d 148
vy319[ajm-1a,d-m::ZF1::mNG knock-in] e 122
vy314[mNG::ajm-1b,c,d,f knock-in] f 104
vy438[ajm-1a,c-m::TagRFP-T knock-in] g 110
vy434[ajm-1a-m::GFP11 knock-in] h 179
0
20
40
60
80
100%
C
ajm-1a-m::GFP11 knock-in;
ptr-10p::GFP1-10
3-fold embryo
Head
hyp TJ
h:g TJ
g:g TJ
D
ajm-1a-m::GFP11 knock-in;
dpy-7p::GFP1-10
3-fold embryo
Head
hyp TJ
hyp TJ

Figure S3

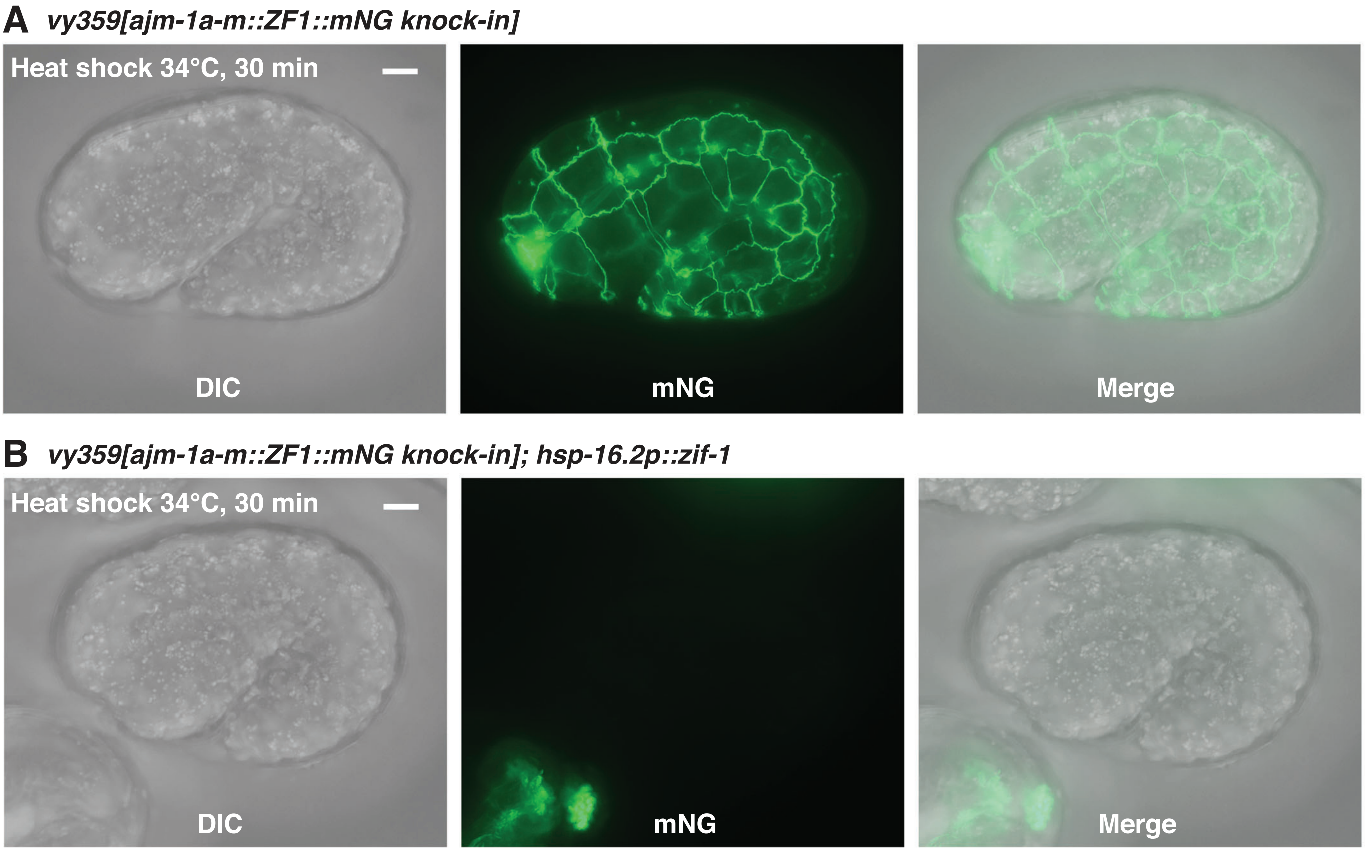
A vy359[ajm-1a-m::ZF1::mNG knock-in]
Heat shock 34°C, 30 min
DIC
mNG
Merge
B vy359[ajm-1a-m::ZF1::mNG knock-in]; hsp-16.2p::zif-1
Heat shock 34°C, 30 min
DIC
mNG
Merge

Figure S4

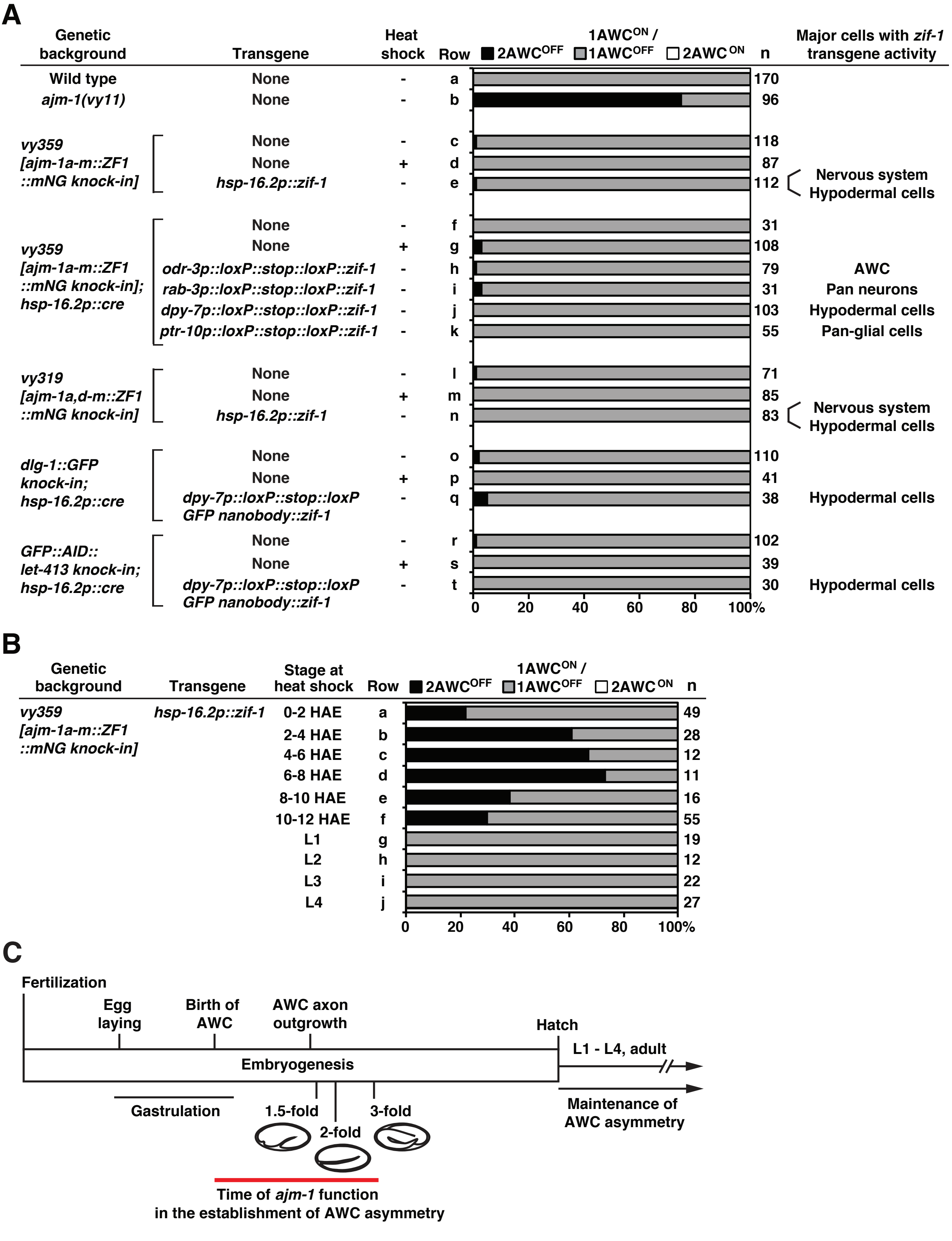

A
Genetic background
Transgene
Heat shock
Row
1AWC^ON / 2AWC^OFF 1AWC^OFF 2AWC^ON
n
Major cells with zif-1 transgene activity
Wild type
ajm-1(vy11)
vy359 [ajm-1a-m::ZF1 ::mNG knock-in]
vy359 [ajm-1a-m::ZF1 ::mNG knock-in]; hsp-16.2p::cre
vy319 [ajm-1a,d-m::ZF1 ::mNG knock-in]
dlg-1::GFP knock-in; hsp-16.2p::cre
GFP::AID:: let-413 knock-in; hsp-16.2p::cre
None
hsp-16.2p::zif-1
odr-3p::loxP::stop::loxP::zif-1
rab-3p::loxP::stop::loxP::zif-1
dpy-7p::loxP::stop::loxP::zif-1
ptr-10p::loxP::stop::loxP::zif-1
dpy-7p::loxP::stop::loxP GFP nanobody::zif-1
a b c d e f g h i j k l m n o p q r s t
170 96 118 87 112 31 108 79 31 103 55 71 85 83 110 41 38 102 39 30
Nervous system Hypodermal cells
AWC
Pan neurons
Hypodermal cells
Pan-glial cells
0 20 40 60 80 100%
B
Genetic background
Transgene
Stage at heat shock
Row
vy359 [ajm-1a-m::ZF1 ::mNG knock-in]
hsp-16.2p::zif-1
0-2 HAE
2-4 HAE
4-6 HAE
6-8 HAE
8-10 HAE
10-12 HAE
L1
L2
L3
L4
a b c d e f g h i j
49 28 12 11 16 55 19 12 22 27
0 20 40 60 80 100%
C
Fertilization
Egg laying
Birth of AWC
AWC axon outgrowth
Hatch
L1 - L4, adult
Embryogenesis
Gastrulation
1.5-fold
2-fold
3-fold
Maintenance of AWC asymmetry
Time of ajm-1 function in the establishment of AWC asymmetry

Figure S5

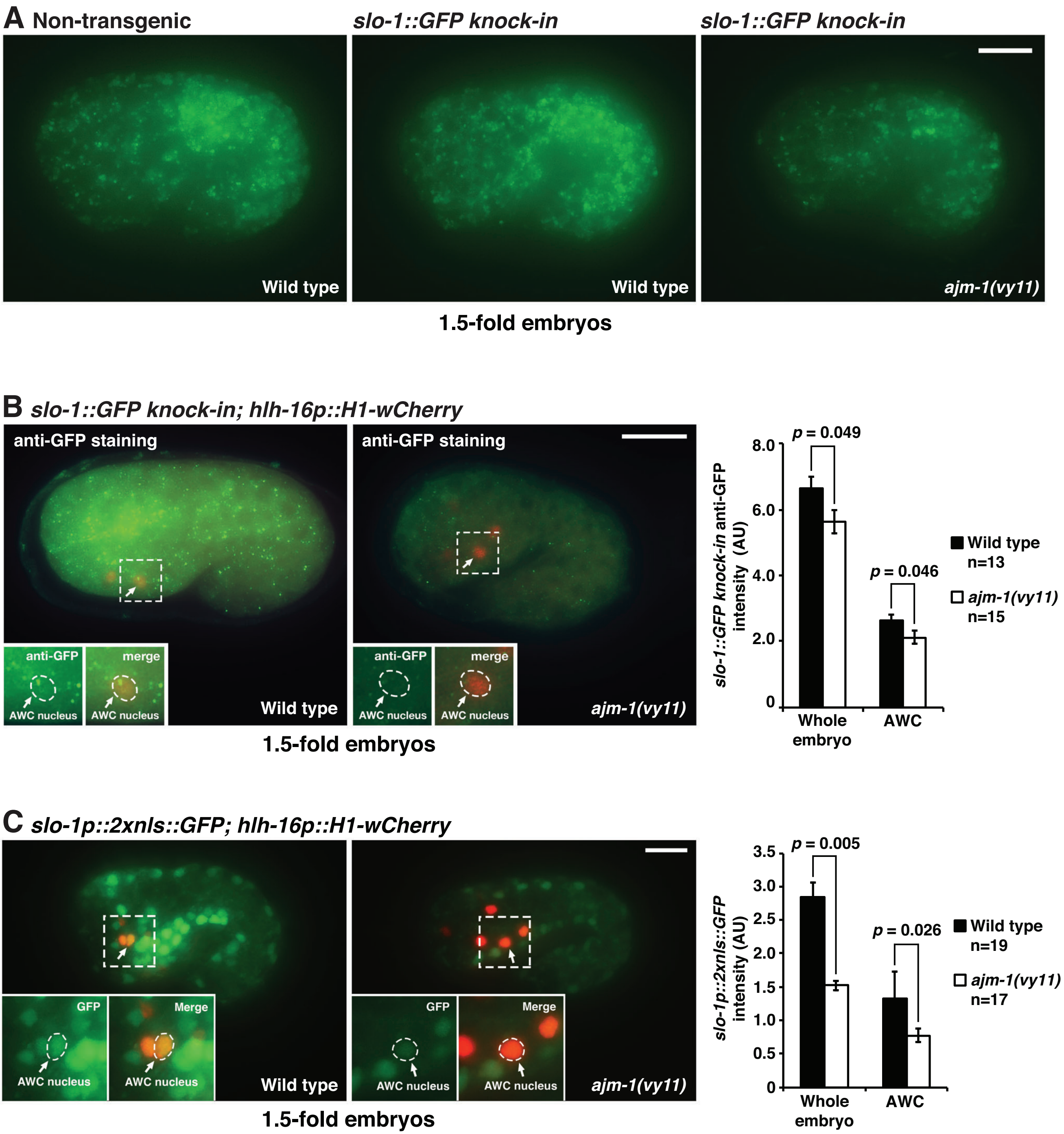
A Non-transgenic
slo-1::GFP knock-in
slo-1::GFP knock-in
Wild type
Wild type
ajm-1(vy11)
1.5-fold embryos
B slo-1::GFP knock-in; hlh-16p::H1-wCherry
anti-GFP staining
anti-GFP staining
anti-GFP
merge
AWC nucleus
AWC nucleus
Wild type
anti-GFP
merge
AWC nucleus
AWC nucleus
ajm-1(vy11)
1.5-fold embryos
slo-1::GFP knock-in anti-GFP intensity (AU)
8.0
6.0
4.0
2.0
0
p = 0.049
p = 0.046
Whole embryo
AWC
Wild type n=13
ajm-1(vy11) n=15
C slo-1p::2xnls::GFP; hlh-16p::H1-wCherry
GFP
Merge
AWC nucleus
AWC nucleus
Wild type
GFP
Merge
AWC nucleus
AWC nucleus
ajm-1(vy11)
1.5-fold embryos
slo-1p::2xnls::GFP intensity (AU)
3.5
3.0
2.5
2.0
1.5
1.0
0.5
0
p = 0.005
p = 0.026
Whole embryo
AWC
Wild type n=19
ajm-1(vy11) n=17

Figure S6

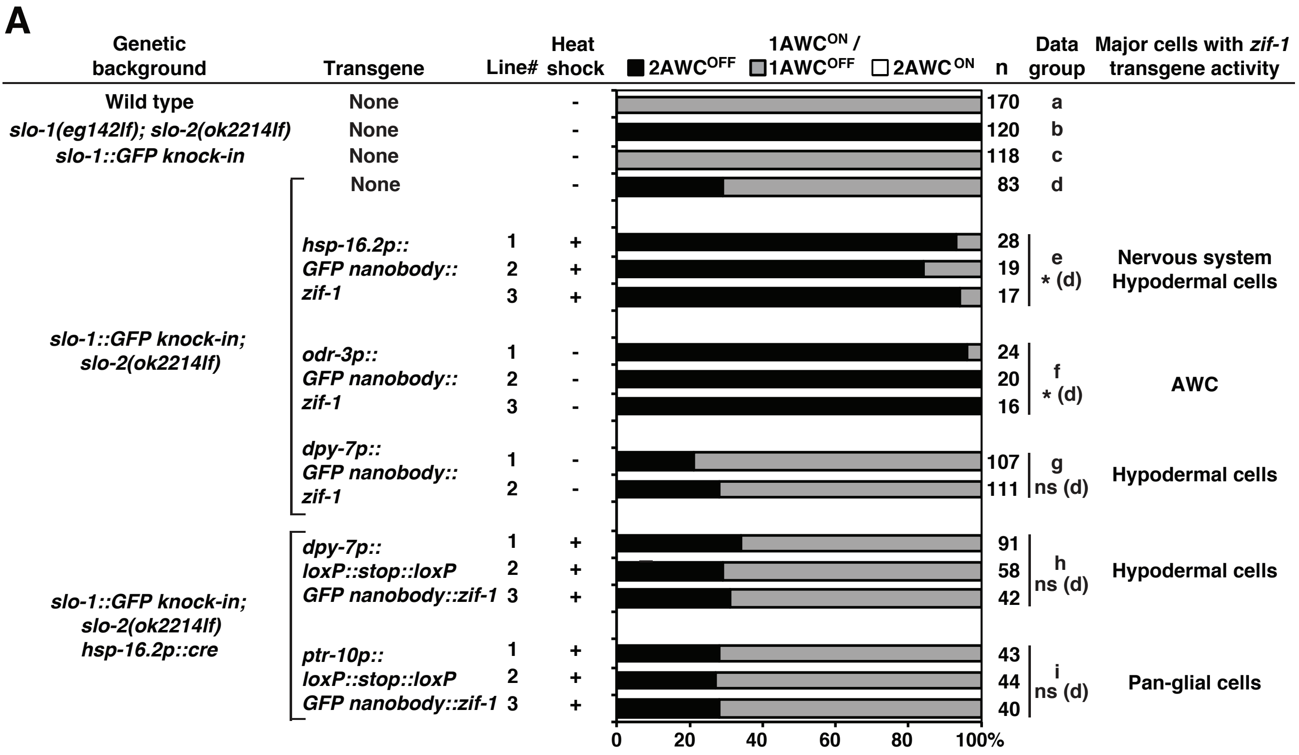
A
Genetic background
Transgene
Line#
Heat shock
2AWCOFF
1AWCON / 1AWCOFF
2AWCON
n
Data group
Major cells with zif-1 transgene activity
Wild type
None
slo-1(eg142lf); slo-2(ok2214lf)
slo-1::GFP knock-in
slo-1::GFP knock-in; slo-2(ok2214lf)
hsp-16.2p:: GFP nanobody:: zif-1
odr-3p:: GFP nanobody:: zif-1
dpy-7p:: GFP nanobody:: zif-1
slo-1::GFP knock-in; slo-2(ok2214lf) hsp-16.2p::cre
dpy-7p:: loxP::stop::loxP GFP nanobody::zif-1
ptr-10p:: loxP::stop::loxP GFP nanobody::zif-1
170 a
120 b
118 c
83 d
28 19 17 e * (d)
24 20 16 f * (d)
107 111 g ns (d)
91 58 42 h ns (d)
43 44 40 i ns (d)
Nervous system
Hypodermal cells
AWC
Hypodermal cells
Hypodermal cells
Pan-glial cells
0 20 40 60 80 100%

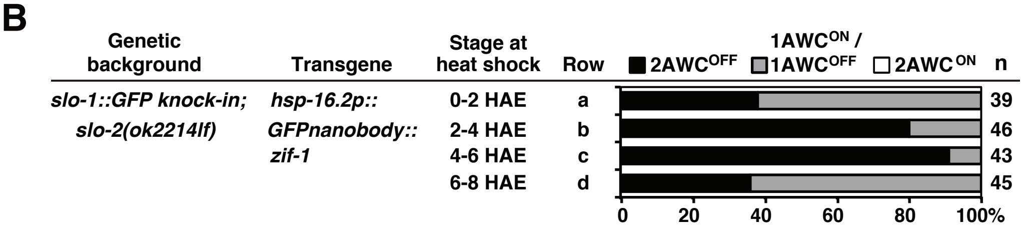
B
Genetic background
Transgene
Stage at heat shock
Row
2AWCOFF
1AWCON / 1AWCOFF
2AWCON
n
slo-1::GFP knock-in; slo-2(ok2214lf)
hsp-16.2p:: GFPnanobody:: zif-1
0-2 HAE a 39
2-4 HAE b 46
4-6 HAE c 43
6-8 HAE d 45
0 20 40 60 80 100%

Figure S7

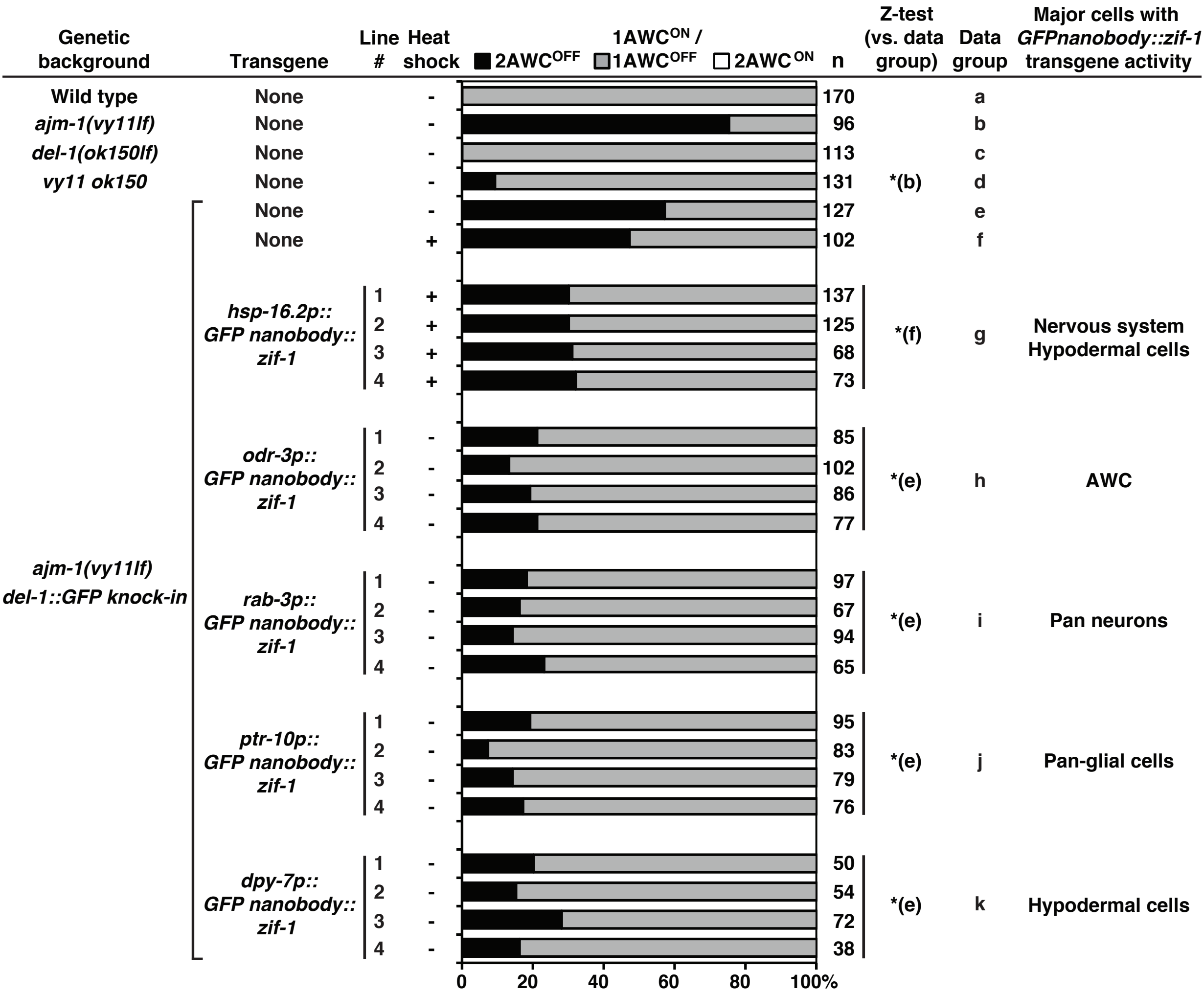
Genetic background
Transgene
Line #
Heat shock
2AWC^OFF
1AWC^ON / 1AWC^OFF
2AWC^ON
n
Z-test (vs. data group)
Data group
Major cells with GFPnanobody::zif-1 transgene activity
Wild type
ajm-1(vy11lf)
del-1(ok150lf)
vy11 ok150
ajm-1(vy11lf) del-1::GFP knock-in
None
hsp-16.2p:: GFP nanobody:: zif-1
odr-3p:: GFP nanobody:: zif-1
rab-3p:: GFP nanobody:: zif-1
ptr-10p:: GFP nanobody:: zif-1
dpy-7p:: GFP nanobody:: zif-1
1
2
3
4
-
+
170
96
113
131
127
102
137
125
68
73
85
102
86
77
97
67
94
65
95
83
79
76
50
54
72
38
*(b)
*(f)
*(e)
a
b
c
d
e
f
g
h
i
j
k
Nervous system Hypodermal cells
AWC
Pan neurons
Pan-glial cells
Hypodermal cells
0
20
40
60
80
100%
